# Gut microbiota and obesity: New insights

**DOI:** 10.3389/fnut.2022.1018212

**Published:** 2022-10-14

**Authors:** Yoredy Sarmiento-Andrade, Rosario Suárez, Beatriz Quintero, Kleber Garrochamba, Sebastián Pablo Chapela

**Affiliations:** ^1^School of Medicine, Universidad Técnica Particular de Loja, Loja, Ecuador; ^2^Department of Health Sciences, Universidad Técnica Particular de Loja, Loja, Ecuador; ^3^Departamento de Bioquímica Humana, Facultad de Ciencias Médicas, Universidad de Buenos Aires, Buenos Aires, Argentina; ^4^Nutritional Support Team, Hospital Británico de Buenos Aires, Buenos Aires, Argentina

**Keywords:** obesity, gut, microbiota, microbiome, overweight

## Abstract

Obesity is a pathology whose incidence is increasing throughout the world. There are many pathologies associated with obesity. In recent years, the influence of the microbiota on both health and pathological states has been known. There is growing information related to changes in the microbiome and obesity, as well as its associated pathologies. Changes associated with age, exercise, and weight changes have been described. In addition, metabolic changes associated with the microbiota, bariatric surgery, and fecal matter transplantation are described. In this review, we summarize the biology and physiology of microbiota in obese patients, its role in the pathophysiology of several disorders associated, and the emerging therapeutic applications of prebiotics, probiotics, and fecal microbiota transplantation.

## Introduction

Chronic disease conditions such as cancer, hypertension, type 2 diabetes (T2D), and obesity are well known as common causes of disease burden worldwide. Obesity is often the initial pathophysiological mechanism of these pathologies and is currently defined for adults by the World Health Organization (WHO) as a body mass index (BMI) ≥30.0 kg/m^2^ or a waist-hip ratio of more than 0.90 for men and more than 0.85 for women (1, 2). Additionally, underweight is defined as having a body mass index (BMI) < 18.5 kg/m^2^, and overweight as having a BMI between 25.0 and 29.9 kg/m^2^ inclusive (1, 2). Overweight/obese people comprise more than 2.1 billion of the worldwide population (3). Although BMI might be considered an approximate guide because it may not correspond to the same degree of obesity in different individuals, it is the same for both sexes and all ages of adults, thus, it provides the best measure at the population-level, of overweight and obesity. However, when defining overweight and obesity for children, age needs to be considered (4). Studies that have compared BMI with other measures of adiposity have found that, at higher BMI classifications, using BMI gives similar results to other approaches, such as dual-energy x-ray absorptiometry (5).

Pathogenesis of obesity is multifactorial, but one of the most interesting factors being studied during recent decades is the influence of gut microbiota. It has been suggested that some issues related to the gut microbiome, such as its composition, diversity index, relative levels, and functional pathways, may predispose adults toward obesity (6). Microbiota is defined as the community of microorganisms in a specific habitat, and the microbiome as its function in that environment (7). This includes a collection of trillions of microorganisms interacting with human hosts, with effects ranging from beneficial to pathogenic (7). One of the first hypotheses about this association between microbiota and BMI proposed that certain groups of bacteria were efficient in absorbing nutrients and energy and then, through rapid metabolism of nutrients, boosting calories absorbed, leading to an increase in BMI (8). It must be considered that overgrowth of bacteria of the phylum *Firmicutes*, accompanied by reduction of bacteria from phylum *Bacteroidetes*, was a characteristic of obese mice and human intestines (9). However, recent data has not confirmed the differences in the Bacteroidetes/Firmicutes ratio between lean and obese humans (9–11). This issue suggests instead another implicated mechanism, such as the high number of bacteria belonging to the *Bacteroidetes* phylum that significantly affects glucose intolerance caused by the consumption of a high-fat diet (9). Other proposed ways of microbiota contributing to obesity are through the anorexigenic gut GLP-1 and, especially, dysregulation of bile acid (BA) signaling mediated by gut microbiota, which could be a promising strategy for obesity therapy (9, 12). The aim of this review is to elucidate recent reports associating gut microbiota with obesity.

## Changes in the microbiota in the obese

Due to massive sequencing techniques (shotgun sequencing), it has been possible to identify the profile of the intestinal microbiota and how its composition affects human metabolism, playing a fundamental role in the development of this disease (12–14). Several studies have observed a difference in the number of bacteria in obese subjects compared to people with normal weight and although 90% of the intestinal microbiota is composed of *Firmicutes* and *Bacteroides*, there is controversy regarding the relative abundance of these bacteria and their causal relationship (15).

### Microbiota, obesity, and age

**Figure 1:**
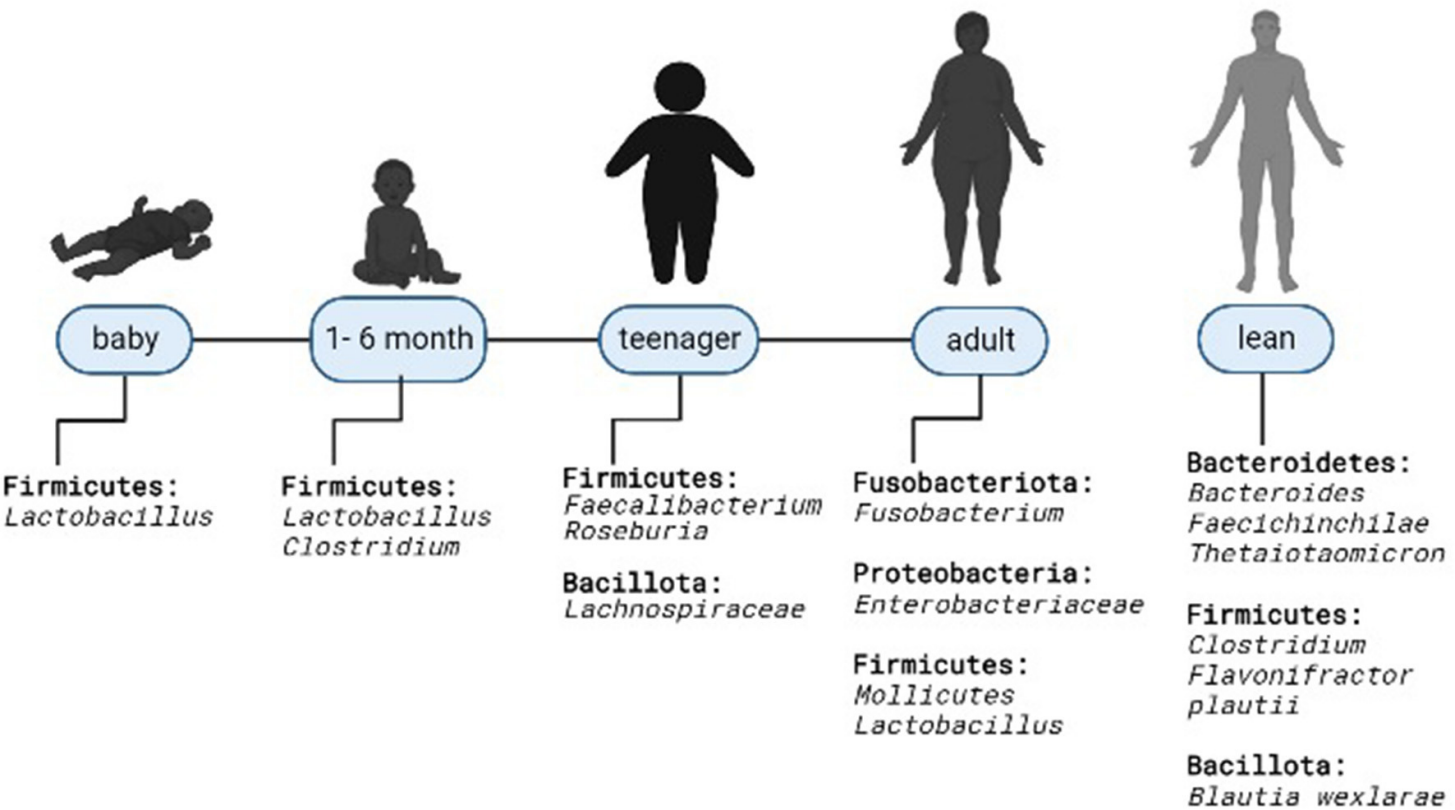
Microbiota modification in overweight and obese humans. Predominant microbiota composition at the phylum and genus level in relation to thin.

States of overweight and obesity are most often observed in increasingly younger populations, including gestational obesity, which may be associated with the composition of the intestinal microbiota (Figure 1) (16). Gestational obesity modifies the intestinal microbiota, with elevated levels of *Bacteroides* in the third trimester; this condition doubles the risk of neonatal obesity due to changes in the microbial composition of the infant's intestine. This is demonstrated by the population in a cohort study of 935 mother-baby couples where 7.5% of newborns of obese mothers presented obesity at 1 year (OR 3.80; 95% CI 1.88–7.66) and 3 years of age (OR 3.79; 95% CI 2.10–6.84); these children had a greater wealth of *Firmicutes P* < 0.05 (17). Although it is not yet clearly defined, it is known that the microbiota increases during the first year of life and is modified with age (16). As confirmed in the study with 12 twins whose microbiota composition was measured and it was observed that both normal weight and obese children had a predominance of *Veillonella, Klebsiella Akkermansia, Streptococcus, or Staphylococcus* at 1 month of age while at 6 months *Bifidobacteria, Lachnospiracea incertae sedis, Escherichia and Shigella* predominated without a significant difference in terms of diversity (*P* > 0.05) and richness of bacterial species of these groups (*P*-value for Chao index was 0.849) (16).

Also, microbial compositions were identified in 96 female adolescents (14–19 years) divided into three groups: eutrophic (EUT) with adequate body fat percentage (BF%), high EUT and BF%, and obese with high BF%, where the last group had a predominance of *Firmicutes* (median 5.5 IQ 2.5–10.5) with respect to the other groups, without statistical significance (*P* = 0.384) (14).

### Microbiota, obesity, and weight

The relationship between microbiota and body weight control has been investigated, without a specific causality or association between these two factors (18), and it is thought that the composition of bacterial species varies according to the presence or not of obesity (14). In Iran, the composition of gut bacteria of 50 normal-weight people was compared with 50 obese people, and it was shown that patients with obesity had an increase in the *Firmicutes/Bacteroides (F/B)* ratio (*p* = 0.002) (19). The same relationship was observed in 61 adult individuals from Ukraine, where the *F/B* ratio increased as BMI values increased (OR = 1.23; 95% CI 1.09–1.38) (18). Similarly, in a Chinese population of children aged between 3 and 18 years, it was identified that the relative abundance of *F/B* was significantly higher in obese people compared to non-obese (20). These results are contrasted with the observational study of 163 healthy young people, divided into three groups (overweight, normal weight, and low weight), which did not show significant differences in the phylum (*p* = 0.55), family (*p* = 0.10) or gender (*p* = 0.12) and in all three *Firmicutes* and *Bacteroidetes* predominated (11). Although, this study demonstrated the higher abundance of the genus *Firmicutes* in the overweight group compared to low weight group (*P* = 0.002), and diversity was significantly lower in the group with the overweight (*P* = 0.007 for Shannon index and *P* = 0.009 for the Ace index) and low weight (*p* = 0.05 for the Shannon index and *p* = 0.08 for the Ace index) (11). Similarly, in the study of the intestinal bacterial composition of 1 and 6 months old lactating twins, there was also no significant difference in the diversity of species between subjects of normal weight and obese (*P* > 0.05); even in 1-month-old infants with normal weight there was a greater abundance of *Lactobacillus* and in the obese *Rhomboutsia* predominated and in the obese 6 months old, *Clostridum sensu stricto* (16).

### Microbiota obesity and bile acids

The intestinal microbiota acts in the metabolism of BA through processes of deconjugation and dehydroxylation in the intestinal lumen (21). They transform primary BA into secondary: cholate into deoxycholate and chenodeoxycholate into lithocolate (LCA) (22). This action is due to bile salt hydrolase enzymes present mainly in the phyla of *Firmicutes and Bacteroidetes*, especially in the genera Clostridium clusters (22).

On the other hand, in the enterocytes of the colon, the BA that will be reabsorbed binds to the farnesoid X receptors. This action stimulates the production of fibroblast growth factor-19 (FGF19) and decreases the hepatic synthesis of BA; in addition, this BA activates the G protein-coupled plasma membrane bile acid receptor (TGR5) by increasing the production of GLP-1. This hormone modulates glucose homeostasis, energy metabolism (23, 24), and the synthesis, conjugation, and transport of BA (21). When the enzymatic action of the microbiota is altered, the composition of the BA does so, facilitating the absorption of fat and causing obesity (12). This was corroborated in a recent study evaluating 183 subjects with high BMI (121 metabolically healthy and 62 metabolically unhealthy), the unhealthy obese subjects had a significantly lower proportion of secondary BA compared to primary ones (OR 1.129, IC 95%: 1.083–1.176, *P* < 0.01); being a predictor in healthy subjects with high BMI (AUC = 0.87, IC 95%: 0.82–0.93, *P* < 0.01, a cut-off value of 66.1 with a sensitivity of 78.5% and a specificity of 91.9%) suggesting that the altered composition of BA may be involved in different metabolic states of obesity (12).

In parallel, a study was conducted on rodents to identify changes in BA metabolism and its association with the gut microbiota, where the group of rodents fed with high-fat diets was divided into a group prone to obesity and another group resistant to it (12). It was observed that the composition of the microbiota did not vary significantly in both groups. However, the genus was abundant in *Clostridium scindens* and *Clostridium hylemonae* in rodents prone to obesity. Due to their high capacity for bioconversion, these bacteria modify the metabolism of the BA and promote obesity. This conclusion was reaffirmed by findings in this same group of rodents of a decrease in secondary BA and an increase in primary ones (12).

### Microbiota, obesity, and exercise

The microbiota can be modified with physical activity, improving the metabolic profile and immune response, an already demonstrated effect in animal and human studies (25). These temporary modifications differ between normal weight and individuals with obesity. This difference was demonstrated in 32 subjects (18 thin and 14 with obesity) who, after performing physical activity, the microbiota composition showed a variation of the genus of bacteria with a predominance of *Bacteroides* in those with obesity and *Faecalibacterium and Lachnospira* in normal weight subjects (26). Also, showed that microbiota composition differed from pre-exercise, and these changes remitted after exercise cessation (26). Similar results were observed in 27 sedentary obese people who, after moderate and intense physical activity, reduced the *F/B* ratio (*P* = 0.04) with an increase in *Bacteroides* (*p* =0.03) and a reduction in *Blautia* (*P* = 0.05) and *Clostridium* (*P* = 0.04) (27). Similarly, the research on 40 premenopausal women with BMI 20–25Kg/m^2^ (19 active and 21 sedentary) did not detect significant differences in alpha and beta diversity or the *Firmicutes*/*Bacteroidetes* relationship (*p* = 0.115) between the two groups. Nevertheless, there was more presence of *Firmicutes* (*p* = 0.085) and lower presence of *Bacteroidetes* (*p* = 0.076) in active women (28).

## Microbiota and pathologies associated with obesity

**Figure 2:**
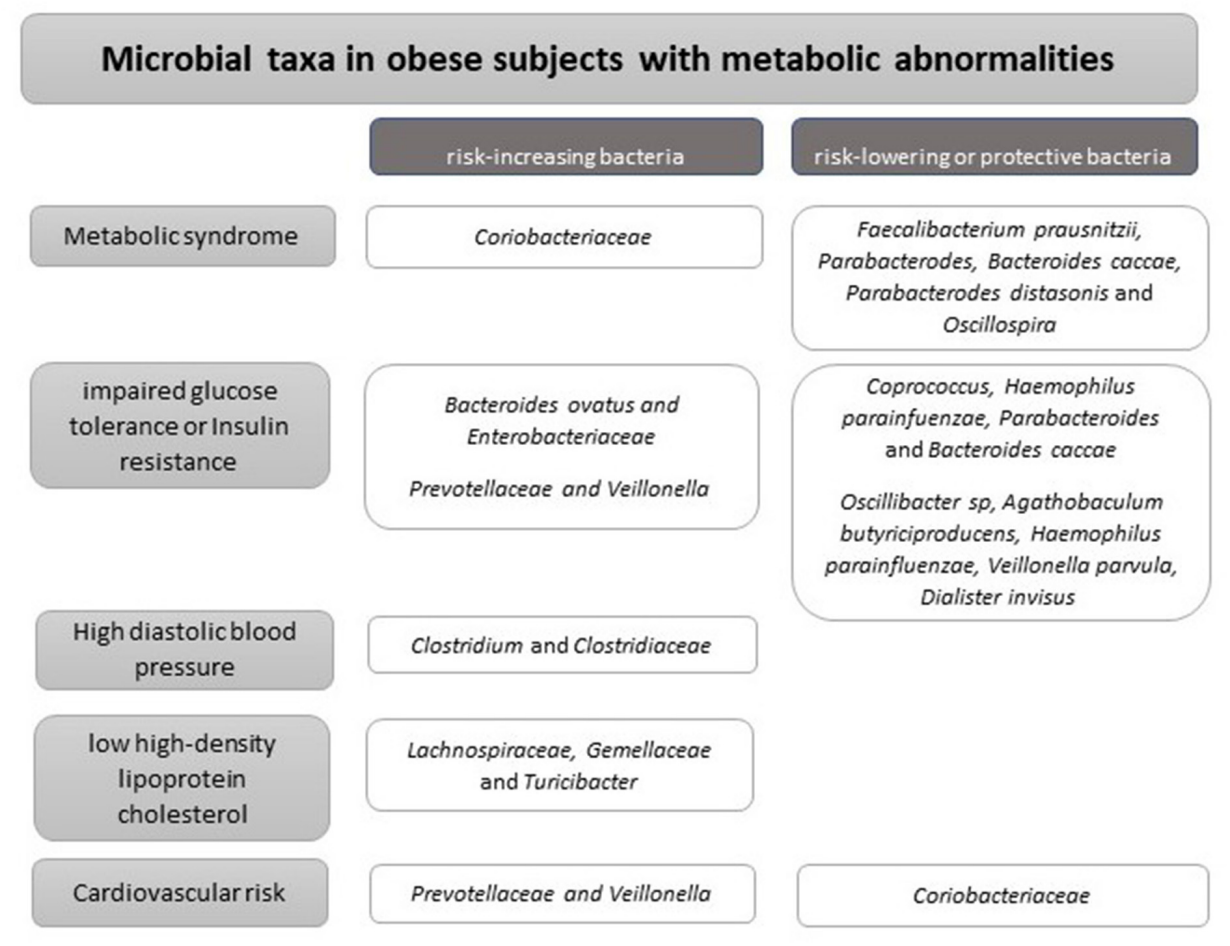
Microbial taxa in obese subjects with metabolic abnormalities.

Obesity is a source of chronic low-level inflammation in various tissues, associated with metabolic defects (29), such as glucose intolerance, insulin resistance (30–32), and cardiovascular diseases (Figure 2) (33–38). An important risk factor for metabolic defects associated with diabetes, metabolic syndrome, and cardiovascular disease is inflammation. The interaction between the host and a certain microbiota pattern has been related to a different profile of interleukin expression (39). For example, lower levels of anti-inflammatory cytokines, including IL-4, IL-13, and IL-10, have been found in obese subjects with metabolic syndrome compared to those without metabolic syndrome (40).

On the other hand, the gut microbiota can influence glucose metabolism and host hormone regulation by producing various metabolites, such as short-chain fatty acids and BA (41–43). Furthermore, elevated levels of bacterial lipopolysaccharide (LPS) have been found among subjects with obesity induced by a high-fat diet (44, 45). Changes in the intestinal microbiota cause an increase in intestinal porosity, an immune response of the mucosa, and, consequently, an increase in intestinal permeability. It appears that the microbiota releases LPS but favors their translocation, possibly leading to metabolic endotoxemia and insulin obstruction (44, 45). Meanwhile, hyperglycemia increases intestinal permeability causing the translocation of some bacterial products, such as LPS, to the systemic circulation, which contributes to chronic inflammation of the liver and adipose tissue associated with impaired glucose or insulin resistance as well as metabolic syndrome (46).

A matter of great importance is that bacterial LPS has been reported not to be necessary for impaired glucose or insulin tolerance (47). The association between bacterial LPS and insulin resistance was reported in gnotobiotic mice, where the authors found that the presence of gut bacteria caused impaired glucose metabolism and increased accumulation and polarization of macrophages toward the pro-inflammatory M1 phenotype in white adipose tissue and, consequently, the development of insulin resistance (47). Although the gut microbiota induced LPS-dependent macrophage accumulation in white adipose tissue, the altered systemic glucose metabolism was not LPS-dependent (47). These results also indicate that macrophage accumulation in white adipose tissue does not always correlate with impaired glucose metabolism (47).

On the other hand, in 68 children with obesity, patients with insulin resistance and higher blood pressure had reduced bacterial richness. Also, different associations were found between higher pressure levels and the presence of *Clostridium and Clostridiaceae*, lower LDL cholesterol and *Lachnospiraceae, Gemellaceae, Turicibacter*, higher metabolic syndrome risk, and the presence of *Coriobacteriaceae* meanwhile *Faecalibacterium prausnitzii, Parabacterodes, Bacteroides caccae, Oscillospira, Parabacteroides distasonis, Coprococcus, and Haemophilus parainfluenzae* were associated lower metabolic syndrome score, fasting glucose, and HOMA_IR (48). A cross-sectional non-controlled study in 21 children with obesity found no significant differences in the abundance of bacterial phyla across different insulin resistance profiles, but a lower microbiome diversity related to children with higher insulin resistance (49). Interestingly, the decrease in microbiome diversity was more pronounced in obese children with a higher degree of insulin resistance (49). Consonantly, a cross-sectional study on normal-weight, overweight adults and those with obesity found that younger subjects have lower inflammation, lower total and visceral adiposity, and lower levels of indicators linked to cardiovascular risk and insulin resistance (50). In that study, subjects with high inflammation scores were associated with a reduced microbiota diversity and bacterial taxa as the family *Prevotellaceae* and the genus *Veillonella*, in contrast, the family *Coriobacteriaceae* seemed to exert a protective effect against the proinflammatory phenotype (50).

Furthermore, some specific bacterial species are associated with a lower level of insulin resistance in the context of obesity. In a nematode, the supplementation with *Pediococcus acidilactici* neutralized the effect of high glucose levels, apparently by modulation of the insulin/IGF-1 signaling pathway (51). Similarly, in obese and lean Asian subjects, a negative correlation has been found between the intestinal abundance of *Akkermansia muciniphila* and the risk of increased severity of insulin resistance (52).

## Microbiota, weight loss, and metabolic changes

The microbiota is maintained thanks to an appropriate intestinal environment, although its survival can be modified by dietary composition, lifestyle, and the use of prebiotics, probiotics, and antibiotics. This understanding offers a new approach to treating metabolic alterations (53). As has been pointed out, the gut microbiota plays a role in the pathogenesis of obesity and associated diseases, and research about interventions with probiotics, prebiotics, and synbiotics (prebiotic and probiotic components) is ongoing to evaluate if these interventions could correct the alteration of the microbiota in obesity and inflammation (54–56).

Dysbiosis, given by the interruption of the healthy symbiotic relationship of the microbiota with the intestine, has been proposed as a contributing factor to obesity and its consequences: T2D, non-alcoholic fatty liver disease (NAFLD), cardiovascular disease (CVD), and cancer (57, 58). This dysbiosis increases the release of LPS, an endotoxic molecule of the outer membrane of Gram-negative bacteria, which alters immunity and the intestinal mucosal barrier causing “leaky gut” and inflammatory pathways activation (59). Marel Roberfroid defined Prebiotics in 1995 (60) as “a selective ingredient that allows changes in composition and activity in the intestinal microbiota and confers wellbeing and health in the host” (60). Prebiotics are non-digestible fibers and can be fermented by gut microbiota (61). Fructans and arabinoxylans (dietary fiber) are the most studied. The intestinal microbiota ferments them into short-chain fatty acids (SCFAs: acetate, propionate, butyrate), CO2, H+, CH4, and other metabolites that regulate metabolic processes (62). Several studies have found contradictory effects on metabolic parameters, describing both neutral (63–65) and positive effects (66–68), and another one has shown influence on the reduction of inflammatory parameters (69). These randomized controlled trials have been developed including, different populations of obese, pre-diabetic, and T2D people.

Most probiotics belong to species with similar functions to the symbiotic microbiota. Probiotics have presented the ability to hydrolyze bile salts, reduce fat accumulation, and reduce systemic inflammation and leptin values, in addition to negatively regulating the expression of “peroxisome proliferator-activated receptor-y (PPAR-y)” at the hepatic level; however, more research is required (70). Research has shown similar controversy with prebiotics. Łagowska and Drzymała-Czyz, in an randomized controled trial (RCT) with overweight/obese women with polycystic ovary syndrome (PCOS), found no additional beneficial effects on SCFA levels, selected gut bacteria abundance, or lipid profile (71). Another study on overweight and obese pregnant Australian women found that the probiotics used (*Lactobacillus rhamnosus* and *Bifidobacterium animalis subspecies lactis*) did not prevent gestational diabetes mellitus (GDM) (72). On the other hand, in Korean obese individuals, a *Lactobacillus plantarum* K50 (LPK) supplementation for 12 weeks significantly reduced the total cholesterol and triglyceride levels with an increase in *L. plantarum* and a decrease in *Actinobacteria*, both correlated with changes in visceral adiposity (73). In postmenopausal women, the effects of a multi-species probiotics intervention altered the influence of microbiota on biochemical, physiological, and immunological parameters without affecting its diversity and taxonomic composition (74).

A recent study found an association between a decrease in blood glucose over time and an increase in *Lactobacillus* abundance with a symbiotic supplement. However, the decrease in BMI, waist circumstance, and body fat mass was associated with a decrease in *Bifidobacterium* abundance over time, supporting the idea that the synbiotic supplement used in this clinical trial modulates human gut microbiota by increasing the abundance of potentially beneficial microbial species (54). Two randomized clinical trials in Thailand have reported an association between gut microbiota and anthropometric and metabolic parameters. The first one compared symbiotic supplementation and placebo groups in Thai obese adults (BMI ≥ 25 kg/m^2^) according to the Asia-Pacific criteria, aged 18–65 years (75). After 12 weeks of supplementation, in the synbiotic group, significant differences (*P* < 0.05) were observed in body weight, BMI, body fat, waist circumference, waist/hip ratio, HDL-C, LDL-C, IL-6, IL-10, IL-1β, TNF-α, IgA, LPS, and zonulin values compared to the baseline values, meanwhile in placebo group no significant changes were obtained (75). In the second study, in older adults, probiotics were indicated. As a result, they achieved an improved intestinal barrier function (up to 48%), a significant increase in the high-density lipoprotein cholesterol (HDL-C), better obesity-related anthropometric biomarkers, and an improvement of short-chain fatty acid levels in human subjects (76). Another study reported that specific metabolite changes following synbiotic intervention might suggest some advantage in providing *Bifidobacterium lactis* in combination with fructooligosaccharide in a low-energy diet, rather than probiotics or diet alone (77).

The clinical effects on body fat regulation seem more significant when probiotics are administered. The intervention combining the probiotic (B420) with prebiotic (refined ultra polydextrose or LU) in overweight and obese adult patients compared to B420, LU, demonstrated that the gut microbiota was modified for all groups compared to placebo (78). The 6-month intervention showed that the group of patients receiving B420 had a higher abundance of *Akkermansia* (*P* < 0.01) and *Streptococcus* (*P* < 0.01) compared to the placebo (78). The intervention with B420+LU increased the abundance of *Akkermansia, Christensenellaceae*, and *Methanobrevibacter*, while there was a reduction of *Paraprevotella*. This species correlates positively with fat body mass and suggests that its increase in abundance may be detrimental to metabolic health. In addition, a negative correlation of the *Family Christensenellaceae* with waist-hip index, energy consumption, and hip body fat mass was demonstrated (78).

### Bariatric surgery

Currently, bariatric surgery is considered the only sustainable, efficient treatment for obesity (79). Surgical procedures such as Roux-Y Gastric Bypass (RYGB) and Sleeve Gastrectomy (SG) facilitate a 50–70% decrease in body weight and fat mass (79). In addition, it leads to a decrease in caloric intake or malabsorption and metabolic changes, improves glucose metabolism, and produces changes in gut microbiota (79).

The role of altered host-microbiota interactions is not fully understood. A significant association has been shown between changes in the microbiota and clinical markers of patients undergoing bariatric surgery (RYGB, SG) (79). The increase in bilirubin is associated with the increase in *Prevotellaceae, Bacteroidales*, and *Peptococcaceae* taxa; the increase in iron is associated with an increase in *Pasteurellaceae*; the decrease in HbA1c is associated with a decrease in *Coriobacteriacea* and the increase in *Clostridiales* taxa. The most pronounced positive association is described between *Lachnospiraceae* and *Coriobacteriaceae* taxa in the reduction of cholesterol levels; however, the associations described correspond to the sequential impact of a crash diet followed by RYGB and SG, in which progressive weight loss and changes in the composition of the microbiota of patients with morbid obesity have been demonstrated (79). The changes in the microbiota that persist after surgery suggest an anatomical and physiological adaptation, as well as reduced acid production, elevated oxygen content, and altered concentration of bile acids. The effects of the crash diet are associated with an increase in *Bifidobacteriaceae* and a decrease in *Streptococcaceae*, while the effect of surgery shows an increase in *Streptococcaceae* and a decline in *Bifidobacteriaceae* (79).

**Figure 3:**
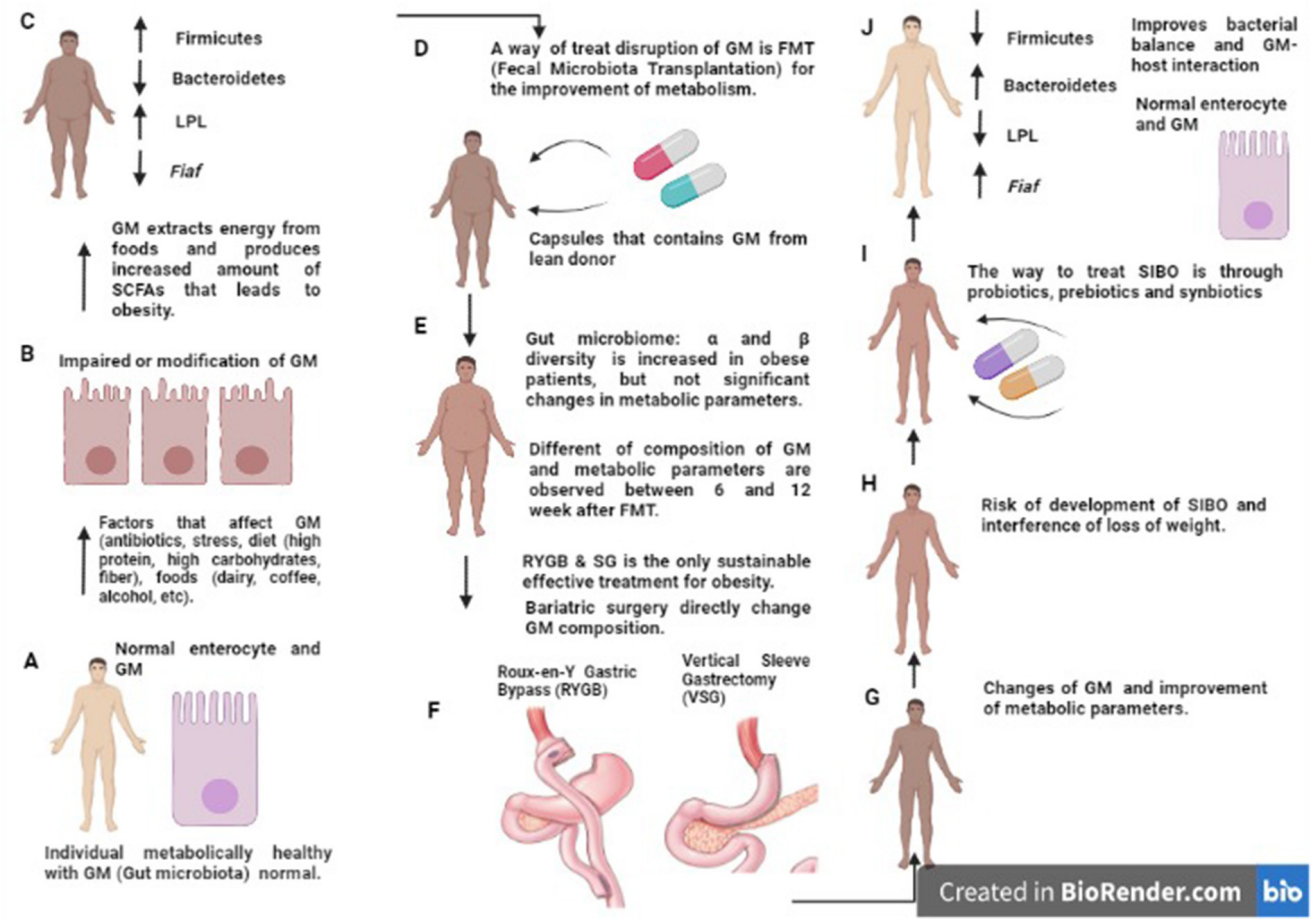
Disruption in intestinal microbiota composition and treatment by FMT, BS, probiotics, prebiotics and synbiotics. Schematic representation of altered GM composition in obesity and MS. In people metabolically healthy, the GM and enterocyte are normal **(A)**. In obesity, altered GM composition (diet, drugs, stress, etc.) inhibits Fiaf in epithelial cells and increases LPL activity; this leads to increased synthesis of triglycerides, fatty acids, and fat storage in adipocytes **(B,C)**; the opposite effect can be seen after treatment (RYGB, VSG) **(D–F)** in which the amount of Fiaf inhibits LPL [described in **(J)**]. However, there is a risk of SIBO **(G,H)**. Prebiotics, probiotics, and synbiotics are an alternative to treatment and have been described to achieve a balanced gut microbiota and lead to a healthy metabolic state **(I,J)**. MS, Metabolic Syndrome; GM, Gut microbiota; Fiaf, Fasting-Induced Adipose Factor or Angptl4; LPL, Lipoprotein lipase; RYGB, Roux-en-Y Gastric Bypass; VSG, Vertical Sleeve Gastrectomy; SIBO, Small Intestinal Bacterial Overgrowth.

The crash diet causes changes in the diversity and composition of the microbiota, while surgical procedures (RYBG, SG) prevent early changes in the composition and restoration of the microbial diversity that probably contributes to weight loss (79). The elevated pH resulting from RYGB has been shown to ensure the survival of probiotic bacteria, making surgical patients a therapeutic target for probiotic therapy (80). In addition, patients undergoing these surgical procedures may develop small intestinal bacterial overgrowth (81), a condition that interferes with weight loss and increases the risk of micronutrient deficiency (vitamins and essential elements) that appears to impair and affect the configuration and composition of the intestinal microbiota (Figure 3) (82, 83). Specific interventions to correct the microbial balance and improve microbiota-host interactions are necessary after surgery (84). One of these strategies is probiotic therapy which contributes to weight loss, reduction of small intestinal bacterial overgrowth (SIBO), improvement of the synthesis of micronutrients, and optimization of the metabolic state (Figure 3) (85).

Roux-en-Y Gastric Bypass (RYGB) surgery improves plasma glucose with a long-lasting effect in patients withT2D. Although the mechanism is unclear, RYGB increases the secretion of GLP-1 and improves insulin resistance (86). Obese patients with type 1 and 2 diabetes mellitus (T1D, T2D) undergoing bariatric surgery [RYGB, Omega Loop, sleeve gastrectomy (SG)] have a very different microbiome composition compared to patients with a “normal” microbiome, and the more weight they lose, the more the microbiome differs (Figure 3), and in general, the metabolic status of patients improves significantly (BMI, glucose, HbA1c, triglycerides, etc.) (87). While the effects of bariatric surgery on obesity are widespread, there are also adverse effects: nausea, vomiting, infections, neuropathy due to nutritional deficiency, and eating disorders (depression, anxiety) (88). Regarding BS, gastric banding (GB), RYGB, and microbiota, there is a decrease in *Firmicutes* (Figure 3) and an increase in *Proteobacteria* and *A. muciniphila*. The latter seems to be associated with weight reduction and insulin sensitivity in patients with severe obesity, despite a certain level of abundance of *A. muciniphila* may be required for metabolic benefits (89).

### Restrictive diet

The adequate caloric restriction (CR) advised for patients with metabolic alterations and healthy adults imply a reduction of 40% of the daily recommendation and allows for the observation of changes in the microbial composition, patients with *Prevotella* enterotypes exhibit a significant weight reduction compared to individuals enterotype *Bacteroides* (89). CR can delay the development of metabolic alterations and is associated with changes in the composition and metabolic function of the microbiota (90). A study conducted on overweight and obese women suggests that the intervention with CR of 800 Kcal has allowed achieving a reduction in body weight, adiposity, hyperglycemia, microbial diversity, and abundance of SCFAs effective in weight reduction (91). In addition, the microbiota after CR (post-CR) was characterized by a lower capacity in the extraction of energy from the diet (91). Despite the benefits of the very-low-calorie diets (VLCDs), metabolic alterations could reverse when normal consumption of calories is restarted (92). This configuration of the microbiota is known as the “yo-yo effect”, and the mechanisms of this phenomenon have yet to be investigated (93). In addition, some modifications in the composition of the microbiota are not normalized even if the reduction of body weight has been achieved (93). A study about the effect of CR in women with obesity described that the intervention of 4 weeks of CR led to a significant weight loss and also reduced systemic inflammation, and improved the integrity of the intestinal barrier, instead a microbial alteration could be due to a high protein or high gluten diet (94).

## Fecal microbiota transplantation

Another current hypothesis about the usefulness of the gut microbiota in weight loss is the one that refers to the possible regulation of glycemia and body weight through the manipulation of the intestinal microbiota (Figure 3). Previous studies have shown that fecal microbiota transplantation (FMT) from lean individuals to individuals with obesity and metabolic syndrome transiently reduced peripheral insulin sensitivity, among other metabolic outcomes: total cholesterol (−0.6 mmol/l), HbA1c (−0.2%), and plasma glucose (−0.6 mmol/l) (95). Donor and recipient microbiomes can vary in graft diversity and composition, so neither all of them are effective, nor are all recipients responsive to microbiome therapy (95). Future research should explore whether donor and recipient preselection or specifically designed microbial composition can optimize changes in the microbiota and whether the use of the microbiome in conjunction with exercise and diet could synergistically improve metabolism in patients with obesity (95).

Fecal microbiota transplantation from lean donors to patients with obesity and metabolic syndrome (MS) has shown significant improvement in insulin sensitivity after 6 weeks with FMT from a lean donor (allogeneic) vs. FMT autologous or aFMT (96). In addition, aFMT based on self-administration of microbiota from a beneficial state to an altered state has been shown to induce rapid recovery from antibiotic-produced dysbiosis (97). aFMT is also associated with attenuation in weight regain, and administration of aFMT capsules is associated with long-term weight maintenance (98). This personalized treatment is considered an alternative to allogeneic transplantation, has greater efficacy, and presents fewer side effects (99). It is described that when trying to relate FMT, diet, and exercise in mice, effects such as reducing fat mass and food efficacy intake may be transmissible *via* FMT and suggest a therapeutic potential for treating individuals with obesity. FMT has also shown efficacy in the treatment of ulcerative colitis (UC). It has been described that after 1 week of treatment with anaerobic FMT from a donor, it results in a high probability of remission at 8 weeks (100). Still, if FMT can transmit beneficial effects of diet and exercise to alter metabolic profiles has not yet been investigated (101).

## Conclusion

Some GM processes have been better studied through intensive research that associates GM with obesogenic mechanisms. One is related to its participation in the BA's metabolism that finally contributes, in different ways, to increase the GLP-1 production, leading to more efficient glucose homeostasis, decreased insulin resistance, reduced fat absorption, and weight gain risk.

Another way that GM may affect bodyweight control is through its effects depending on modifications to other metabolic processes related to immune function, mainly due to elevated levels of bacterial LPS, which have been found among subjects with obesity induced by a high-fat diet. These changes might cause an immune response of the mucosa and, consequently, an increase in intestinal permeability, even though it is not LPS dependent. Otherwise, hyperglycemia may also increase intestinal permeability, causing the translocation of LPS to the systemic circulation, which contributes to chronic inflammation of the liver and adipose tissue associated with the development of impaired glucose or insulin resistance, as well as metabolic syndrome.

Studies have been conducted about the influence of physical activity (PA) on GM to improve body weight control. PA improves the metabolic profile and immune response, at least temporarily, probably by incrementing *Bacteroidetes* proportion in GM.

Nevertheless, there are still many gaps in the underlying mechanisms that GM exerts around body weight control. One poorly understood mechanism is related to how specific GM composition and function are associated with body weight dysregulation and related pathologies. Even though most of the research indicates that the ratio of *Firmicutes/Bacteroides* is higher in the obese gut, other researchers indicate the opposite or no association with this ratio.

On the other hand, there is still much controversy regarding the benefits, dosage, ideal composition, time of administration, side effects, and feasibility studies concerning pre-pro and symbiotic use in humans, as well as the real benefits of FMT in the prevention and management of obesity. Nonetheless, most research has found a more significant benefit in using symbiotics than prebiotics or probiotics administered individually. Besides, the aFMT has been associated with attenuation in weight regain and administration of aFMT capsules with long-term weight maintenance, but assessment and focus on individual response to intervention are needed.

Therefore, despite the evidence supporting that GM induces favorable changes in the intestine of the subject with obesity, research on maintaining the long-term effects of therapeutics for the prevention and treatment of metabolic disorders associated with obesity is still lacking.

## Author contributions

RS, YS-A, BQ, and KG wrote the paper. SC conceived the draft, supervised the writing, and corrected the manuscript. All authors contributed to the article and approved the submitted version.

## Conflict of interest

The authors declare that the research was conducted in the absence of any commercial or financial relationships that could be construed as a potential conflict of interest.

## Publisher's note

All claims expressed in this article are solely those of the authors and do not necessarily represent those of their affiliated organizations, or those of the publisher, the editors and the reviewers. Any product that may be evaluated in this article, or claim that may be made by its manufacturer, is not guaranteed or endorsed by the publisher.

## Abbreviations

BA: Bile Acid
BF%: Body Fat Percentage
BMI: Body Mass Index
CR: Caloric Restriction
CVD: Cardiovascular Disease
EUT: eutrophic
FIAF: Fasting-Induced Adipose Factor
F/B: *Firmicutes/Bacteroides*
GM: Gut Microbiota
LPS: Lipopolysaccharide (LPS)
NAFLD: Non-Alcoholic Fatty Liver Disease
PPAR-y: Peroxisome Proliferator-Activated Receptor-y
RYGB: Roux-Y Gastric Bypass
SCFAs: Short-Chain Fatty Acids
SG: Sleeve Gastrectomy
SIBO: Small Intestinal Bacterial Overgrowth
T2D: Type 2 Diabetes
VSG: Vertical Sleeve Gastrectomy
WHO: World Health Organization.
